# Multiple Field-of-View Based Attention Driven Network for Weakly Supervised Common Bile Duct Stone Detection

**DOI:** 10.1109/JTEHM.2023.3286423

**Published:** 2023-06-15

**Authors:** Ya-Han Chang, Meng-Ying Lin, Ming-Tsung Hsieh, Ming-Ching Ou, Chun-Rong Huang, Bor-Shyang Sheu

**Affiliations:** Department of Computer Science and EngineeringNational Chung Hsing University 34916 Taichung 402202 Taiwan; Department of Internal MedicineNational Cheng Kung University Hospital, College of Medicine, National Cheng Kung University 63461 Tainan 701401 Taiwan; Department of Medical ImageNational Cheng Kung University Hospital, College of Medicine, National Cheng Kung University 63461 Tainan 701401 Taiwan; Cross College Elite Program, and Academy of Innovative Semiconductor and Sustainable ManufacturingNational Cheng Kung University 34912 Tainan 701401 Taiwan

**Keywords:** Common bile duct (CBD) stone detection, choledocholithiasis, weakly-supervised learning, deep learning, object detection

## Abstract

Objective: Common bile duct (CBD) stones caused diseases are life-threatening. Because CBD stones locate in the distal part of the CBD and have relatively small sizes, detecting CBD stones from CT scans is a challenging issue in the medical domain. Methods and procedures: We propose a deep learning based weakly-supervised method called multiple field-of-view based attention driven network (MFADNet) to detect CBD stones from CT scans based on image-level labels. Three dominant modules including a multiple field-of-view encoder, an attention driven decoder and a classification network are collaborated in the network. The encoder learns the feature of multi-scale contextual information while the decoder with the classification network is applied to locate the CBD stones based on spatial-channel attentions. To drive the learning of the whole network in a weakly-supervised and end-to-end trainable manner, four losses including the foreground loss, background loss, consistency loss and classification loss are proposed. Results: Compared with state-of-the-art weakly-supervised methods in the experiments, the proposed method can accurately classify and locate CBD stones based on the quantitative and qualitative results. Conclusion: We propose a novel multiple field-of-view based attention driven network for a new medical application of CBD stone detection from CT scans while only image-levels are required to reduce the burdens of labeling and help physicians automatically diagnose CBD stones. The source code is available at https://github.com/nchucvml/MFADNet after acceptance. Clinical impact: Our deep learning method can help physicians localize relatively small CBD stones for effectively diagnosing CBD stone caused diseases.

## I. Introduction

The presence of gallstones in the common bile duct refers as the common bile duct (CBD) stone which is also known as choledocholithiasis. Most cases of choledocholithiasis result from gallstones stuck in the common bile duct [1]. As shown in [2], up to 20% of gallbladder stone cases are associated with CBD stones. CBD stones caused acute suppurative cholangitis and acute biliary pancreatitis [3] are life-threatening. These diseases should be diagnosed and treated immediately even in asymptomatic ones [4].

The gold standard treatment in managing CBD stones nowadays is endoscopic retrograde cholangiopancreatography (ERCP). However, ERCP is an invasive procedure and may result in about 6.9% to 12% of adverse events even performed by experienced endoscopists [5], [6]. Some adverse events are lethal and needed to be prevented. To preventing ERCP [7], patients suspected of having choledocholithiasis can also be diagnosed by history taking, blood test, physical examination, and ultrasound scanning. However, the positive prediction rates of these tests are ranged from 59% to 64% [8], [9].

Compared with these methods, diagnosing CBD stones from computed tomography (CT) scans achieves better diagnostic accuracy [10], [11]. However, the results are interpreter dependent and the process is time-consuming. Compared with larger gallstones, CBD stones locate in the distal part of the CBD and have relatively small sizes. Thus, they may not be clearly captured by CT scans. Automatically and effectively detecting CBD stones from CT scans becomes a novel and emerging issue in the medical domain. A novel technical solution is expected to address this clinical need in the interdisciplinary field and improve the quality of patient care efficiently.

Recently, supervised convolutional neural networks (CNNs) are widely utilized to solve medical image processing problems [12], [13], [14]. In general, a large number of training data is required for CNNs to learn representative models for object detection. Moreover, object-level labels such as bounding boxes are required for supervised CNNs. These labels bring the time-consuming burdens for physicians and also heavily rely on physicians’ experience.

**FIGURE 1.**
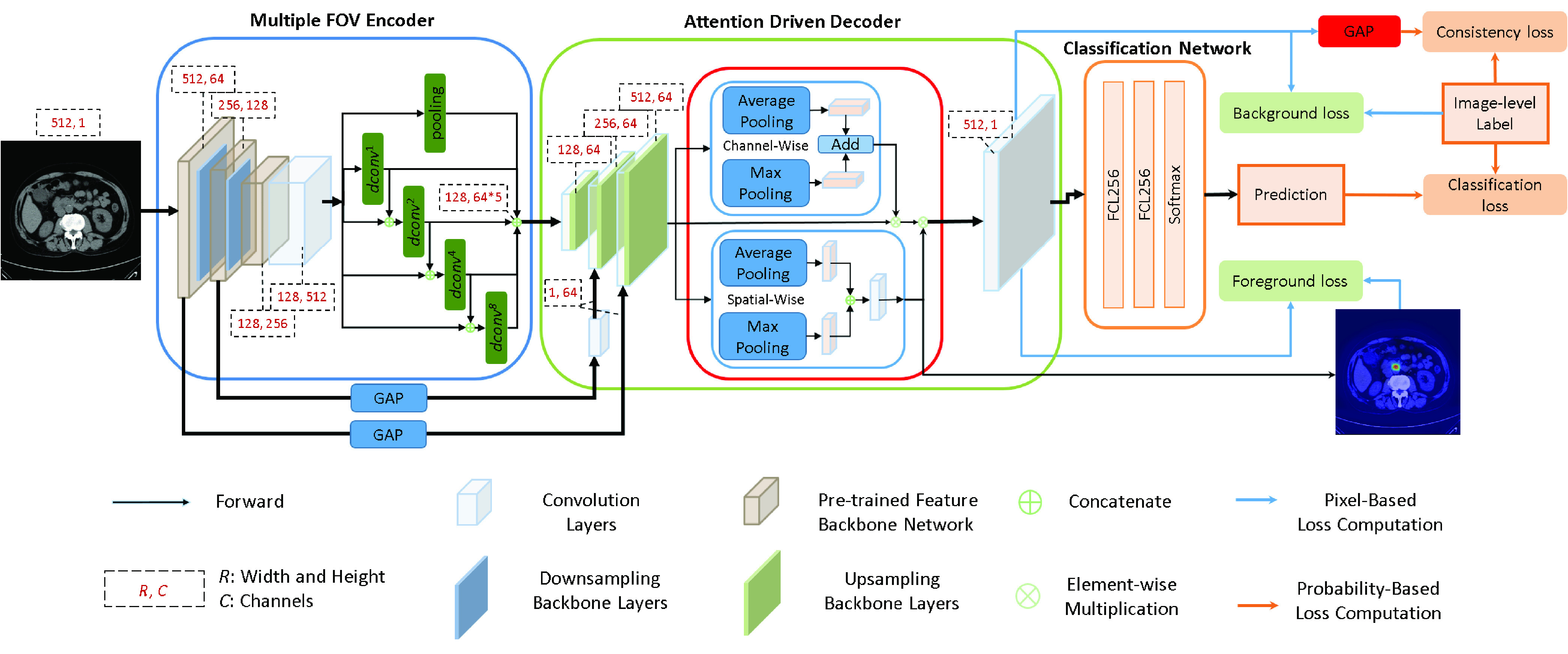
The network architecture of the proposed method. The multiple field-of-view encoder aims to generate deep features of different resolutions to represent CBD stones of different sizes. The attention driven decoder generates the channel attention map, spatial attention map and probability map. The channel attention map enhances the decoder features via effective channel information. The spatial attention map aims to help locate the CBD stones based on image-level labels. The probability map reduces the false detection of CBD stones based on the background loss and serves as the feature for the classification network. The consistency loss ensures that the dominant feature response of the probability map is consistent with the image-level label. Finally, the classification loss drives the predictions of the classification network to classify the CT scan. The dash rectangles show the widths, heights and channels of the features maps.

In this paper, we would like to propose a novel technical method to solve the clinic CBD stone detection problem from CT scans in a weakly-supervised manner, i.e. only the image-level labels are given without the requirement of the ground truth bounding boxes of the CBD stones. To achieve the goal, we propose a novel multiple field-of-view based attention driven network (MFADNet). As shown in Fig. 1, the proposed network is composed of a multiple field-of-view encoder, an attention driven decoder and a classification network. We apply the multiple field-of-view encoder to extract the encoder feature based on the dilated convolutions [15] of different dilated rates. The encoder feature represents the multi-scale contextual information of the CT scan. Through the decoder, the encoder feature is further upsampled to obtain the decoder feature of the higher resolution for CBD stone detection. Via the spatial-channel attention scheme [16] of the decoder, the spatial attention map and channel attention map are generated to represent salient responses of the decoder feature for localizing the CBD stones. By integrating the decoder feature, spatial attention map and channel attention map, the probability map is generated to distinguish CBD stones from normal tissues.

To drive the learning of the network via image-level labels, four losses including the foreground loss, background loss, consistency loss and classification loss are proposed. Here, the foreground loss aims to learn the locations of the CBD stones. It indicates the correlations of the spatial attention map and the probability map based on the image-level labels with CBD stones. The background loss aims to avoid miss-detection of CBD stones for normal CT scans. It drives the probability map to represent normal regions based on the image-level labels without CBD stones. The consistency loss aims to ensure that the learned dominant feature response of the probability map needs to be consistent with the image-level labels. Finally, the classification loss is computed based on the prediction of the classification network to achieve accurate image-level classification. With the combination of these losses, we can train the proposed network with weak labels in an end-to-end training manner. During the inference, the bounding boxes of the detected CBD stones are located by searching the regions of the dominant responses in the spatial attention map. As shown in the experiments, the proposed method can successfully achieve CBD stone detection compared with the state-of-the-art weakly-supervised methods.

There are three main contributions in this paper. First, this is the first deep learning based weakly-supervised method for CBD stone detection from CT scans based on our best knowledge. Our method not only proposes a novel weakly-supervised network for a novel application in the medical domain, but also reduces the burdens of annotations. Second, the attention driven decoder with the proposed foreground loss, background loss and consistency loss helps accurately locate CBD stones by using image-level labels. These losses also drive the end-to-end training of the network. Third, while the class activation map (CAM) based methods [17], [18] aim to obtain salient features based on the learned networks, the proposed method utilizes the aforementioned losses to effectively drive the learning of the features and locations of the CBD stones. Thus, the proposed method achieves outstanding performance compared with state-of-the-art weakly-supervised methods.

The remaining parts of the paper are organized as follows. Sec. II introduces the related weakly-supervised methods. Sec. III presents the proposed method and implementation details. The collected dataset and experimental results are shown in Sec. IV. Finally, the conclusions are given in Sec. V.

## II. Related Work

Although supervised deep learning methods [19], [20], [21], [22], [23], [24] have achieved amazing performance for lesion classification, detection or segmentation in the medical domain, time-consuming annotated labels are required. To reduce the labeling burdens of the physicians, weakly-supervised methods are proposed to achieve computer-aided diagnosis for CT scans based on weakly annotated labels. Many weakly-supervised methods [17], [18], [25], [26], [27] are proposed in the computer vision domain. In the following, we focus on the reviews of the weakly-supervised methods in the medical domain.

Wang et al. [28] proposed a label denoising network (LDnet) to segment male pelvic organs from CT scans with 3-D bounding box labels. Li and Xia [29] proposed a deep reinforcement learning-based method for lymph node segmentation by using two cross line-annotations on the lymph node. They used GrabCut [30] to generate pseudo ground truths for U-Net [19]. Tang et al. [31] proposed an attention enhanced model with a regional level set loss to achieve lesion segmentation.

Due to the COVID-19 issues, many state-of-the-art weakly-supervised methods focus on the detection and segmentation of lesions from chest CT scans. Wang et al. [12] proposed a 3-D deep convolutional neural network for COVID-19 detection from CT scans. They segmented lung regions by using a pre-trained U-Net. Then, a 3-D deep neural network and a class activation map (CAM) [17] were applied to predict the COVID-19 infection and localize the regions. Yang et al. [32] proposed a generative adversarial network (GAN) [33] based weakly-supervised method for COVID-19 lesion localization. They subtracted the output image from the input image to localize lesions. Similar GAN based weakly-supervised method can also be found in [34]. Qian et al. [35] presented a multi-task multi-slice deep learning method with two networks. Their method diagnosed diseases for each single CT scan and generated localization maps of abnormalities, while the patient-level classification network provided the prediction based on the features of the network. Liu et al. [36] proposed using scribble-level annotations for segmenting COVID-19 lung infections. Uncertainty-aware and transformation-consistent schemes were considered to make consistent segmentation results with respect to different perturbations of the CT scan.

Besides CT scans, Madooei et al. [37] proposed using a multiple instance learning (MIL) framework for identifying blue-white structure from dermoscopy images based on image-level labels. Chamanzar and Nie [38] proposed a deep learning method to achieve cell segmentation and detection based on point labels. van Sloun and Demi [39] proposed using a fully convolutional neural network to locate B-lines from ultrasound scans. Ma et al. [40] proposed a multi-scale class activation map (MS-CAM) to solve the weakly-supervised geographic atrophy lesion segmentation problem from spectral-domain optical coherence tomography images. The geographic atrophy lesion is retrieved based on the projection of the segmentation of the MS-CAM. Meng et al. [41] proposed a complementary heatmap-based method to achieve multi-retinal disease detection from fundus images with weakly-supervised labels. Qi et al. [42] proposed a graph-regularized embedding network to model the cross-region and cross-image relationships on chest X-ray images for weakly-supervised disease localization.

Compared with the aforementioned weakly-supervised methods, the proposed method extracts the spatial-channel attention feature from the multiple field-of-view feature to detect CBD stones of different sizes. Then, based on the proposed losses and image-level labels, the proposed method can successfully classify the CT scans and locate the CBD stones in an end-to-end trainable manner. In addition, the proposed method is the first weakly-supervised method to detect CBD stones and has been shown to achieve the state-of-the art performance compared with competing weakly-supervised methods from different research domains.

## III. Proposed Method

In this section, an overview of the proposed method is first presented. Then, the multiple field-of-view encoder, the attention driven decoder and the classification network are described. The losses are introduced to address how can the proposed method effectively locate the CBD stones based on image-level labels. Finally, we provide the implementation details.

### A. Overview

Let a weakly annotated set of CT scans with image-level labels be $D=\{I_{n}, y_{n}\}^{N}_{n=1}$, where $I_{n}$ denotes the $n$th CT scan and $y_{n} \in \{1, 0\}$ is its image label to indicate if $I_{n}$ contains the CBD stone or not, and $N$ is the number of CT scans. The width and height of each CT scan are denoted as $W$ and $H$. With the weakly annotated dataset $D$, we aim to derive a detection model to locate CBD stones in CT scans. Fig. 1 illustrates the proposed network architecture.

The proposed network contains a multiple field-of-view encoder, an attention driven decoder and a classification network. The encoder aims to represent CBD stones by using the multiple field-of-view scheme. Then, the feature of the encoder is refined by the attention driven decoder with the proposed losses to locate CBD stones. The classification network provides the image-level predictions of the CT scans.

During training, we consider a CT scan $I$ and its label $y$, where $I$ serves as the input of the multiple field-of-view encoder. The encoder contains a feature backbone network and a multiple field-of-view network to produce the encoder feature $\boldsymbol {f}_{E}$ of $I$. Then, the attention driven decoder with an upsampling backbone network and a spatial-channel attention network [16] is proposed to locate the CBD stones based on the proposed losses. The feature $\boldsymbol {f}_{U}$ produced by the upsampling backbone network is the input of the spatial-channel attention network. The spatial-channel attention network generates the channel attention map $\boldsymbol {m}_{c} \in \mathbb {R}^{1 \times 64}$ and spatial attention map $\boldsymbol {m}_{s} \in \mathbb {R}^{WH \times 1}$. By fusing $\boldsymbol {f}_{U}$, $\boldsymbol {m}_{c}$ and $\boldsymbol {m}_{s}$ with a $1 \times 1 $ convolutional layer, a probability map $\boldsymbol {m}_{p} \in \mathbb {R}^{WH \times 1}$ is generated to distinguish CBD stones from normal regions.

To enforce the learned spatial attention map $\boldsymbol {m}_{s}$ to locate CBD stones based on the weakly annotated image-level label, $\boldsymbol {m}_{s}$ is applied to compute the foreground loss $\ell _{fg}$ with respect to $\boldsymbol {m}_{p}$. When the training image does not contain the CBD stone, the background loss $\ell _{bg}$ is computed based on $\boldsymbol {m}_{p}$ to avoid the miss-detection of CBD stones of the normal training image. Besides $\ell _{bg}$, to ensure that the spatial prediction of $\boldsymbol {m}_{p}$ can be consistent with the weakly annotated image-level label, a global average pooing layer is applied to $\boldsymbol {m}_{p}$ to compute the consistency loss $\ell _{con}$. Finally, $\boldsymbol {m}_{p}$ is passed to the classification network to obtain the image-level prediction. A classification loss $\ell _{cls}$ is computed based on the prediction and the image-level label $y$. In summary, the whole network is optimized in a weakly-supervised manner by using the following loss function:

\begin{equation*} \ell = \omega _{fg} \ell _{fg} + \omega _{bg} \ell _{bg} + \omega _{con} \ell _{con} + \omega _{cls} \ell _{cls}, \tag{1}\end{equation*}

where $\omega _{fg}$, $\omega _{bg}$, $\omega _{con}$ and $\omega _{cls}$ are the weights of the losses. The network will be described in details in the following.

### B. Multiple Field-of-View Encoder

The multiple field-of-view encoder contains a feature backbone network and a multiple field-of-view network. The feature backbone network is used to extract the deep feature $\boldsymbol {f}_{b}$ to represent $I$. It is a pre-trained convolutional neural network based on the ImageNet dataset [43]. The downsampling layers of the feature backbone network are achieved by $2 \times 2$ max pooling. Because the sizes and spatial context relationship of CBD stones are variant, $\boldsymbol {f}_{b}$ is hard to provide representative deep features to handle the scale problem of CBD stone detection. Thus, we enhance $\boldsymbol {f}_{b}$ by using the multiple field-of-view network to extract features of different resolutions as follows.

As shown in Fig. 1, the multiple field-of-view network contains 4 parallel dilated convolutional layers [15] and a maximum pooling layer to represent features in different resolutions. The first dilated feature $\boldsymbol {f}_{d}^{1}$ is obtained by a $3 \times 3$ dilated convolutional layer of the dilated rate 1. To further extend the discriminability of following learned dilated feature, $\boldsymbol {f}_{d}^{1}$ is concatenated with $\boldsymbol {f}_{b}$ and passed to a $3 \times 3$ dilated convolutional layer of the dilated rate 2 to obtain the second dilated feature $\boldsymbol {f}_{d}^{2}$. Similarly, the third dilated feature $\boldsymbol {f}_{d}^{3}$ and the fourth dilated feature $\boldsymbol {f}_{d}^{4}$ of the dilated rates 4 and 8 are obtained based on $\boldsymbol {f}_{b}$ and the dilated features of previous dilated rates, respectively. The $k$th dilated feature is defined as follows:

\begin{align*} \boldsymbol {f}_{d}^{k}= \begin{cases} \displaystyle dconv^{2^{k-1}}(\boldsymbol {f}_{b}),\quad &k = 1 \\ \displaystyle dconv^{2^{k-1}}(\boldsymbol {f}_{b} \oplus {\boldsymbol {f}_{d}^{k-1})},\quad &k>1 \end{cases} \quad, \tag{2}\end{align*}

where $dconv^{2^{k-1}}(\cdot)$ is a $3 \times 3$ dilated convolutional layer of the dilated rate $2^{k-1}$ and $\oplus $ is the concatenation operator of the backbone feature and the previous dilated feature.

Finally, $\boldsymbol {f}_{b}$ is passed to a $2 \times 2$ max pooling layer to obtain the pooling feature $\boldsymbol {f}_{p}$. By concatenating the dilated features and the pooling feature of the multiple field-of-view network, the obtained encoder feature $\boldsymbol {f}_{E}$ can represent the multi-scale contextual information of $I$ and is defined as:

\begin{equation*} \boldsymbol {f}_{E} = \boldsymbol {f}_{d}^{1} \oplus \boldsymbol {f}_{d}^{2} \oplus \boldsymbol {f}_{d}^{3} \oplus \boldsymbol {f}_{d}^{4} \oplus \boldsymbol {f}_{p}, \tag{3}\end{equation*}

where $\oplus $ is the concatenation operator. The encoder feature then serves as the input of the attention driven decoder for CBD stone detection.

### C. Attention Driven Decoder

The attention driven decoder contains an upsampling backbone network and a spatial-channel attention network to locate the CBD stones based on the proposed losses. The upsampling backbone network upsamples the encoder feature $\boldsymbol {f}_{E}$ to obtain the decoder feature $\boldsymbol {f}_{U}$ which has the same spatial resolution of the input image. Two forward connections from the feature backbone network of the encoder provide features of different resolutions to help obtain better decoder features during upsampling. The encoder features of the first block and second block of the feature backbone network are passed to spatial-wise global average pooling layers to generate the features. To ensure the consistency of the feature dimension, a $1\times 1 $ convolutional layer with 64 channels is used to modify the dimension of the feature of the second block after the global average pooling. These features then serve as the weights to multiply the decoder features of the corresponding blocks of the decoder as shown in Fig. 1.

Instead of considering the feature responses of the low-resolution feature maps, we propose applying the spatial-channel attention network based on the output of the upsampling backbone network to compute the channel attention map $\boldsymbol {m}_{c}$ and spatial attention map $\boldsymbol {m}_{s}$. In this way, the computed attention maps can better represent the locations of the CBD stones in the original resolution. The spatial-channel attention network based on the convolutional block attention module [16] is consisted of a channel attention module and a spatial attention module for feature and loss computation. In our method, the channel attention module aims to extract the channel attention map $\boldsymbol {m}_{c}$ which contains representative information of different channels of $\boldsymbol {f}_{U}$. $\boldsymbol {m}_{c}$ is computed by the addition of the channel features after a spatial-wise max pooling layer and a spatial-wise global average pooling layer. Because effective channel information will be reserved, $\boldsymbol {m}_{c}$ is used as the weight map to enhance important features in $\boldsymbol {f}_{U}$.

The spatial attention module aims to obtain spatial attention map $\boldsymbol {m}_{s}$ which is used to locate the CBD stones. $\boldsymbol {f}_{U}$ is passed to a channel-wise max pooling layer and a channel-wise average pooling layer, respectively, to obtain spatial attention features. These features are concatenated and passed to a $7 \times 7$ convolutional layer $conv^{7}(\cdot)$ to obtain the spatial attention map $\boldsymbol {m}_{s}$ as follows:

\begin{equation*} \boldsymbol {m}_{s} = conv^{7}(pool^{max}_{c}(\boldsymbol {f}_{U}) \oplus pool^{ave}_{c}(\boldsymbol {f}_{U})), \tag{4}\end{equation*}

where $pool^{max}_{c}(\cdot)$ and $pool^{ave}_{c}(\cdot)$ are the channel-wise max pooling and channel-wise average pooling functions. If $I$ contains CBD stones, these two pooling functions help emphasize the responses of the CBD stones.

The channel attention map $\boldsymbol {m}_{c}$ aims to extract representative channel features of $\boldsymbol {f}_{U}$ and is defined as follows:

\begin{equation*} \boldsymbol {m}_{c} = pool^{max}_{s}(\boldsymbol {f}_{U})+pool^{ave}_{s}(\boldsymbol {f}_{U}), \tag{5}\end{equation*}

where $pool^{max}_{s}(\cdot)$ and $pool^{ave}_{s}(\cdot)$ are the spatial-wise max pooling and spatial-wise average pooling functions. These two spatial-wise pooling functions help find representative features for CBD stones from $\boldsymbol {f}_{U}$.

Finally, a probability map $\boldsymbol {m}_{p}$ is computed as follows:

\begin{equation*} \boldsymbol {m}_{p} = conv^{1}(\boldsymbol {m}_{s} \otimes (\boldsymbol {m}_{c} \otimes \boldsymbol {f}_{U})), \tag{6}\end{equation*}

where $conv^{1}(\cdot)$ is a $1 \times 1 $ convolutional layer followed by a sigmoid function, and $\otimes $ is the element-wise multiplication. $\boldsymbol {m}_{p}$ aims to help distinguish CBD stones from backgrounds.

To locate CBD stones, we cooperate $\boldsymbol {m}_{p}$ with $\boldsymbol {m}_{s}$ to compute the foreground loss $\ell _{fg}$ which enforces the spatial attention map to learn the locations of the CBD stones. When $\boldsymbol {m}_{p}$ indicates the low probability of the CBD stones for certain pixels, the spatial attention map should also have low attention responses for these pixels. When a training image contains the CBD stone, the network needs to learn the locations of CBD stones based on the pixel-wise feature responses of $\boldsymbol {m}_{s}$. In other words, the pixels with high feature responses of CBD stones in the spatial attention map $\boldsymbol {m}_{s}$ should have low feature responses of $\boldsymbol {m}_{p}$.

By enforcing the learning of $\boldsymbol {m}_{s}$ to locate the CBD stones with respect to $\boldsymbol {m}_{p}$, the foreground loss $\ell _{fg}$ is defined based on pixel-wise feature responses between $\boldsymbol {m}_{s}$ and $\boldsymbol {m}_{p}$ as follows:

\begin{equation*} \ell _{fg}=\frac {1}{W \times H}\sum _{i=1}^{W}\sum _{j=1}^{H}(1-\boldsymbol {m}_{s}^{(i, j)})\times \boldsymbol {m}_{p}^{(i,j)}, \tag{7}\end{equation*}

where $\boldsymbol {m}_{s}^{(i,j)}$ is the feature response of the pixel at position $(i,j)$ of $\boldsymbol {m}_{s}$, $\boldsymbol {m}_{p}^{(i,j)}$ is the feature response of the pixel at position $(i,j)$ of $\boldsymbol {m}_{p}$. The foreground loss offers an supervisory signal to identify possible locations of CBD stones in the weakly-supervised training process of the network.

Besides the foreground loss to locate CBD stones, it is also important to identify normal CT scans without CBD stones based on image-level labels. To distinguish CBD stones from normal regions, we propose the probability map $\boldsymbol {m}_{p}$. To drive the learning of $\boldsymbol {m}_{p}$ to learn normal regions without CBD stones, we propose the background loss $\ell _{bg}$. The background loss aims to reduce the feature responses of $\boldsymbol {m}_{p}$ when inputting a normal training CT scan. Because we only have the image-level label $y$ of the training CT scan, we define the label $y^{(i, j)}$ of the pixel at position $(i,j)$ of the training CT scan as $y^{(i, j)} = y$. The background loss is then defined as follows:

\begin{equation*} \ell _{bg}=\frac {1}{W \times H}\sum _{i=1}^{W}\sum _{j=1}^{H}(1-y^{(i, j)})\times \boldsymbol {m}_{p}^{(i, j)}. \tag{8}\end{equation*}

If the input training CT scan contains the CBD stone, i.e. $y=1$, it should not be used to learn $\boldsymbol {m}_{p}$ to avoid the incorrect learning of CBD stones as backgrounds. Thus, only the normal training CT scans affect the computation of $\boldsymbol {m}_{p}$. By minimizing $\ell _{bg}$, the network can effectively represent normal training CT scans. When the feature responses of $\boldsymbol {m}_{p}$ are small for normal CT scans, the false detection of CBD stones can be avoided.

Besides the pixel-based foreground loss and background loss, we also propose the consistency loss and classification loss which are probability-based loss functions computed by using the dominant feature responses and the predictions to drive the learning of the network based on global image-level labels. While the probability map $\boldsymbol {m}_{p}$ indicates possible normal background regions and CBD stones of the input CT scans, we would like to ensure that the dominant feature response of $\boldsymbol {m}_{p}$ is consistent with the image-level labels of the input CT scans. If the CT scans are normal, the learned $\boldsymbol {m}_{p}$ should contain feature responses with respect to normal image-level labels. Similarly, when the CT scans contain CBD stones, their dominant feature responses should also be consistent with the image-level labels. To address the dominant feature responses for both cases, we propose the consistency loss $\ell _{con}$ as follows:

\begin{align*} \ell _{con}=-y\log (GAP(\boldsymbol {m}_{p}))+(1-y)\log (1-GAP(\boldsymbol {m}_{p})), \tag{9}\end{align*}

where $GAP(\cdot)$ is the global average pooling layer which represents the dominant feature response of $\boldsymbol {m}_{p}$. By using the consistency loss, each training CT scan produces an extra dominant feature response which needs to be consistent with the ground truth image-level label to optimize the whole network.

Finally, $\boldsymbol {m}_{p}$ is used as the input of the classification network consisting of two fully connected layers followed by a softmax layer. The classification network aims to figure out if CBD stones exist in $I$ or not. To guide the model learning, the classification loss $\ell _{cls}$ is defined as follows:

\begin{equation*} \ell _{cls}=-y\log (\hat {y})+(1-y)\log (1-\hat {y}), \tag{10}\end{equation*}

where $\hat {y}$ is the prediction of the classification network. Based on Eq. (10), we can ensure that the predictions of the classification network are consistent with the ground truth image-level labels. Moreover, it will also drive the whole network to learn proper features.

### D. Implementation Details

The feature backbone network [44] is modified from a pre-trained VGG-16 network [45] by adding dropout layers to VGG blocks to avoid overfitting. The upsampling backbone network is composed of three $3 \times 3$ convolutional layers and one $1 \times 1$ convolutional layer. Each $3 \times 3$ convolutional layer is followed by an instance normalization layer. Finally, the classification network consists of two fully connected layers with 256 neurons and a softmax layer for image-level prediction.

The Keras framework on an Intel Core i7 computer with a 3.7 GHz CPU and RTX 2080 GPU is used to implement the proposed method. We use the RMSProp optimizer to train the model. The parameters of $\rho $ and $\epsilon $ of the RMSProp optimizer are set to 0.9 and $10^{-8}$. The batch size is set to 3. The initial learning rate is set to $10^{-4}$. When the loss of the validation stops improving for 5 epochs, the learning rate will be decreased by a factor of 10. The maximal training epoch is set to 100, and if the loss of the validation stops improving in 10 epochs, the training will end early. The weights of the losses are set to $\omega _{fg} = 1$, $\omega _{bg} =0.5$, $\omega _{con}=1$ and $\omega _{cls}=1$, because we empirically found that suppressing the background loss helps increase the detection rate of the CBD stones. To locate the CBD stones in the CT scans, we first extract the feature map from the spatial-channel attention network. To extract salient regions which reflect locations of CBD stones, we apply the channel-wise average pooling to obtain important channel information of the feature map through the channel dimensions. To avoid false detection of the CBD stone of normal CT scans, we apply the probability map as the weight map. In this way, we can obtain the CBD stone attention map $\boldsymbol {m}_{a}$ as follows:

\begin{equation*} \boldsymbol {m}_{a} = pool^{ave}_{c}(\boldsymbol {m}_{s} \otimes (\boldsymbol {m}_{c} \otimes \boldsymbol {f}_{U}))\otimes (1-\boldsymbol {m}_{p}). \tag{11}\end{equation*}

Here, $\boldsymbol {m}_{a}$ is normalized by the maximal value of $\boldsymbol {m}_{a}$. When the normalized values of pixels are larger than the threshold $th$ (= 0.6) and the classification network predicts that the CBD stone exists in the CT scan, these pixels are considered as pixels with CBD stones. Finally, a bounding box is used to extract detection results based on the largest connected component region which is the same as CAM [17].

## IV. Experimental Results

### A. Experimental Settings

#### 1) Dataset

From January 2018 to August 2019, patients who were clinically suspicious of CBD stones and met 2010 American society for gastrointestinal endoscopy (ASGE) high probability criteria for CBD stones in the National Cheng Kung University Hospital were included. All patients were presented with one of the following conditions: (a) total bilirubin more than 4 mg/dL, (b) total bilirubin level ranged from $1.8-3.9$ mg/dL with a dilated (diameter $> 6$mm) common bile duct on images, (c) presented ascending cholangitis clinically, and (d) abdominal ultrasound revealed CBD stone. After the enrollment, patients with known malignancy or medical implants inside the biliary system that causes obstruction of the CBD were excluded. Patients without pre-treatment abdominal CT scans, adolescent and pregnant women were also excluded from the initial cohort. The dataset is approved by Institutional Review Board, National Cheng Kung University Hospital under B-ER-111-186.

In the experiments, abdomen CT scans near the gallbladder regions of 252 patients were collected. 428 CT scans with CBD stones were selected by the physicians. Because the number of the normal CT scans are significantly more than that of the CT scans with CBD stones, the physicians limited the number of the normal CT scans to be twice the number of CT scans with CBD stones to reduce the data imbalance problem and retain the fact that the number of the normal CT scans is more than that of CT scans with CBD stones. To provide more diversity, the number of selected normal CT scans for each patient was randomly decided by the physicians and thus 856 normal CT scans were randomly selected. As a result, each patient has $\sim 5$ selected CT scans on average. The CT scans of the dataset is divided into 7:3 for the training and testing as the setting in [46], [47], and [48]. The training dataset contains 298 CT scans with CBD stones and 596 normal CT scans. The testing dataset contains 130 CT scans with CBD stones and 260 normal CT scans. Only the image-level labels were applied for a weakly-supervised training manner. The resolution of the CT scans is $512 \times 512$.

We apply accuracy, sensitivity, specificity, F1-score metrics to evaluate the classification performance of the proposed method and state-of-the-art methods. Moreover, to evaluate the weakly-supervised detection performance of CBD stones for each method, the mean intersection over union (mIoU) and the average precision (AP) [49] values were employed, and the ground truth bounding boxes of the testing images were manually labelled by an experienced physician. In addition, we used the same procedure shown in CAM [17] to draw bounding boxes for all of the competing methods.

#### 2) Comparative Baselines

Based on our best knowledge, the proposed method is the first weakly-supervised method for CBD stone detection from CT scans. Thus, we compared our method with four state-of-the-art weakly-supervised learning methods from the computer vision domain and medical domain for the evaluations of CBD stone classification and detection. The first competing method is the class activation map (CAM) [17] which applies the global average pooling on the convolutional feature maps before a fully-connected layer to identify the importance of the image regions for object detection. To provide more general explanations of activation maps in convolutional neural networks, Grad-CAM [18] is proposed by using the gradient information back-propagated to the convolutional layer of interest. To extract activation features by using multiple scale information, MS-CAM [40] is proposed for geographic atrophy lesion detection. While the localization of the CAM based methods is easily affected by salient feature responses, structure-preserving activation (SPA) [50] is proposed to extract object structural information for object detection.

### B. Ablation Study

The results of the ablation study are shown in Table 1. The first row shows the results without (w/o) the multiple field-of-view (M-FOV) network. When the multiple field-of-view information is not considered, the sensitivity significantly drops. Such results show the importance of the multiple field-of-view network to help extract representative deep features. The second row shows the results without the channel attention map $\boldsymbol {m}_{c}$ which represents effective channel information of different channels of the decoder feature. When the decoder feature cannot be enhanced by the learned channel features, the classification accuracy of the network degrades. Moreover, the learned features are hard to represent CBD stones and thus the sensitivity also degrades. Compared with $\boldsymbol {m}_{c}$, the spatial attention map $\boldsymbol {m}_{s}$ aims to learn the locations of the CBD stones based on image-level labels. It is also used to compute the foreground loss $\ell _{fg}$. As shown in the third row of Table 1, without $\boldsymbol {m}_{s}$, the sensitivity significantly drops which indicates the importance of $\boldsymbol {m}_{s}$ and $\ell _{fg}$ to identify CBD stones. Please note that results of the ablation study without $\boldsymbol {m}_{c}$ and $\boldsymbol {m}_{s}$ were obtained by ignoring corresponding terms in Eqs. (4) and (5), respectively. The background loss $\ell _{bg}$ aims to ensure that $\boldsymbol {m}_{p}$ can indicate normal regions when the training CT scan does not contain CBD stones. Without $\ell _{bg}$, the specificity drops compared with the proposed method in the fourth row of Table 1. This result shows that $\ell _{bg}$ helps reduce false detection of CBD stones for normal CT scans. The fifth row shows the results without the consistency loss $\ell _{con}$. Because $\ell _{con}$ aims to ensure that the dominant feature response of $\boldsymbol {m}_{p}$ is consistent with the image-level labels, both sensitivity and specificity of the method without $\ell _{con}$ drop compared with the proposed method. The ablation study shows that the proposed network structure and losses are effective.

**TABLE 1:** Ablation Study

Methods	Accuracy	Sensitivity	Specificity	F1-score
w/o M-FOV	0.7821	0.7154	0.8154	0.6836
w/o $\boldsymbol {m}_{c}$	0.8769	0.7462	0.9423	0.8017
w/o $\boldsymbol {m}_{s}$	0.8692	0.7308	0.9385	0.7884
w/o $\ell _{bg}$	0.8744	0.8000	0.9115	0.8093
w/o $\ell _{con}$	0.8718	0.8000	0.9077	0.8062
Ours	0.9051	0.8692	0.9231	0.8593

### C. Quantitative Results

Two kinds of the quantitative performance are compared. The first one is the image-based classification performance shown in Table 2. Compared with the CAM based methods, SPA considers a restricted activation module during object localization to avoid the affections of local extremely high responses in CAM. Thus, SPA achieves a better specificity compared with CAM based methods. The proposed method further considers the foreground loss, background loss and the consistency loss. Thus, it can achieve high sensitivity compared with SPA.

**TABLE 2:** Classification Results Compared with Competing Methods

Methods	Accuracy	Sensitivity	Specificity	F1-score
CAM	0.8179	0.8077	0.8231	0.7473
Grad-CAM	0.8128	0.7308	0.8538	0.7224
MS-CAM	0.8154	0.8462	0.8000	0.7534
SPA	0.8846	0.8077	0.9231	0.8235
Ours	0.9051	0.8692	0.9231	0.8593

The second quantitative performance is to evaluate the weakly-supervised detection results of each method. When the methods can correctly locate CBD stones in the weakly-supervised manner, the detected regions should overlap the ground truth regions. In the CAM based methods, they do not consider to learn the locations of CBD stones but only consider the local extremely high responses of the feature maps. SPA contains a self-correlation map generating module which can improve the attention map based on the structural information to better locate target objects. Because CBD stones are usually inconspicuous and may not contain self-correlations in CT scans, these competing methods fail to locate CBD stones based on their salient feature responses in the weakly-supervised manner. Thus, the area of intersection between the detected regions of these methods and the ground truth regions is very small which leads to significantly low mIoU and AP values of these methods as shown in Table 3. The visualization results in Sec. IV-D also indicate that the detected regions of these methods fail to overlap the ground truth regions. In contrast, the proposed method can achieve better mIoU and AP values under the guidance of the spatial-channel attention network with the proposed losses.

**TABLE 3:** Detection Results Compared with Competing Methods

Methods	mIoU	AP
CAM	0.0206	0.0263
Grad-CAM	0.0011	0.0002
MS-CAM	0.0127	0.0108
SPA	0.0015	0.0017
Ours	0.5309	0.5707

Table 4 shows the confusion matrix of the proposed method. Most CT scans are correctly classified to show that the proposed method can distinguish the CBD stone cases from normal cases. In addition, the average inference time of the proposed method is 0.067 seconds to show the potential of the real-time usage of the proposed method.

**TABLE 4:** The Confusion Matrix of the Proposed Method

	Predict-Positive	Predict-Negative
Real-Positive	113	17
Real-Negative	20	240

### D. Qualitative Results

**FIGURE 2.**
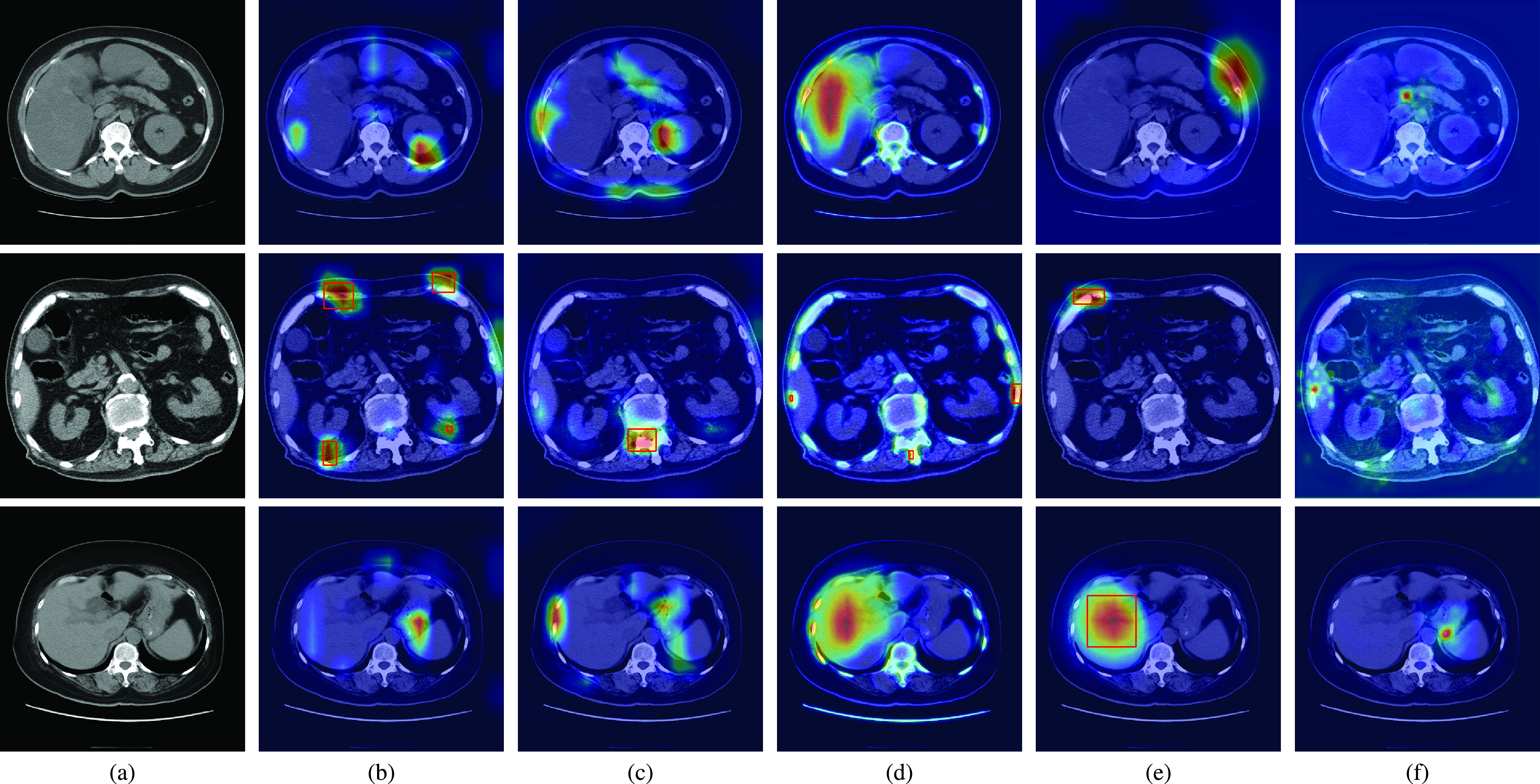
The CBD stone detection results with attention maps for normal CT scans. (a) Ground truth, (b) CAM, (c) Grad-CAM, (d) MS-CAM, (e) SPA, and (f) the proposed method. The red rectangles indicate the false detection results of the CBD stones.

Fig. 2 shows the qualitative results of the state-of-the-art methods and the proposed method for normal CT scans of three patients. The attention maps of each method are also shown in Fig. 2. The red rectangles show the CBD stone detection results when the method incorrectly classifies the normal CT scans as the abnormal CT scans with CBD stones. Fig. 2(a) shows the ground truth of normal CT scans. The results of CAM are shown in Fig. 2(b). Because CAM considers the feature responses after global average pooling, the learned features then easily focus on bone and angiosteosis regions which are salient compared with other organs in the CT scans. Similar results can also be observed in Grad-CAM as shown in Fig. 2(c). As a result, the false detection and false classification results of CAM and Grad-CAM are observed from the CT scan of the second patient.

Compared with CAM and Grad-CAM, MS-CAM considers the scale information with the attention mechanism of the fully connected operations. It can then observe liver regions as shown in the CT scans of the first and third patients of Fig. 2(d). Nevertheless, MS-CAM still incorrectly detects CBD stones for the second patient due to the salient features of bones. As indicated by SPA, these CAM based methods easily miss object structure information because extremely high feature responses are considered. To solve the problem, a restricted activation module is proposed in SPA. Nevertheless, extremely high feature responses such as bone and liver regions still affect the classification results of SPA for the second and third patients as shown in Fig. 2(e). Thus, SPA also incorrectly locates the bone and liver regions as CBD stones. Fig. 2(f) shows the results of the proposed method. With the foreground loss, background loss and consistency loss, the learned attention maps focus on the regions which can distinguish CBD stones from ambiguous regions such as bone regions of the second patient and angiosteosis regions of the third patient. Thus, the proposed method can successfully classify these CT scans as normal CT scans based on the learned features.

**FIGURE 3.**
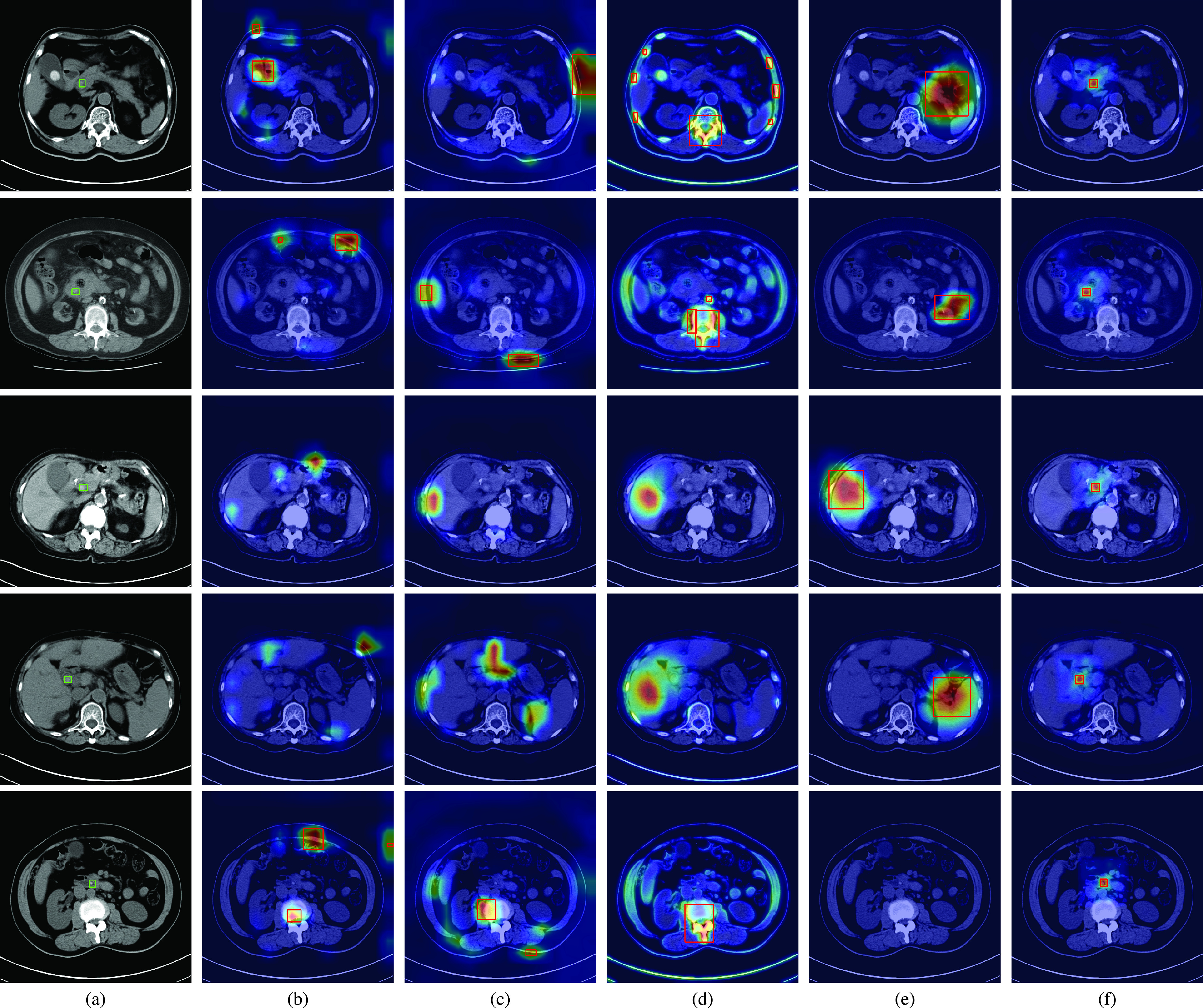
The CBD stone detection results with attention maps for CT scans with CBD stones. (a) Ground truth, (b) CAM, (c) Grad-CAM, (d) MS-CAM, (e) SPA, and (f) the proposed method. The red rectangles indicate the false detection results of the CBD stones.

Fig. 3(a) shows the ground truth regions of CBD stones in CT scans by using green rectangles. The results of CAM, Grad-CAM, MS-CAM, SPA and the proposed method are shown in Figs. 3(b), (c), (d), (e), and (f), respectively. The same as the observations in Fig. 2(b), the salient feature responses of CAM are affected by bone regions for the second and fifth patients, and the gallstones of the first patient. Although CAM successfully classifies the CT scans of the first, second, and fifth patients as CT scans with CBD stones, the locations of detected CBD stones are incorrect compared with the ground truth. Because the CBD stones of the third and fourth patients are not clearly captured by CT scans, the learned salient features of CAM cannot represent the CBD stones for classification. Thus, miss-detection of CBD stones occurs for these two patients. Similar results can also be observed for Grad-CAM and MS-CAM in Fig. 3(c) and (d).

Compared with CAM based methods, SPA considers the restricted activation module to learn local object structure and self-correlation to refine localization maps. Thus, the salient features of SPA can focus on non-bone regions. Nevertheless, SPA still fails to correctly locate CBD stones compared with the ground truth for the first four patients as shown in Fig. 3(e). Because CBD stones are relatively small and are not clearly captured in CT scans, the modules of SPA are hard to learn local object structure of CBD stones from only image-level labels. As a result, the learned features of SPA cannot represent CBD stones for the fifth patient.

As shown in Table 3, the competing methods have low mIoU and AP values. Such results can be visually explained by the salient feature responses of these methods shown in Fig. 3(b), (c), (d) and (e), respectively. These competing methods focus on regions without CBD stones compared with the ground truth regions. Thus, the mIoU values of these methods are naturally low because the detected regions do not overlap the ground truth regions. In contrast, the visualizations shown in Fig. 3(f) reveal that the proposed method provides an interpretable AI model which truly focuses on CBD stones. The cooperation of losses and the whole network provides a novel weakly-supervised learning way to learn salient features to represent CBD stones. Thus, the mIoU and AP values of the proposed method are significantly better than those of the competing methods.

## V. Conclusion

In summary, we propose a novel multiple field-of-view based attention driven network for a new medical application of CBD stone detection from CT scans. Different from CAM based methods, the proposed method is composed of a multiple field-of-view encoder, an attention-driven decoder and a classification network. While the encoder learns representative multiple field-of-view features from CT scans, the decoder learns the locations of CBD stones based on the spatial-channel attention network with the proposed foreground loss, background loss and consistency loss from image-level labels. The classification network provides the image-level prediction. By the guidance of the proposed losses, the network is end-to-end trainable in a weakly-supervised manner. Also shown in the experimental results, CBD stones can be accurately detected and located compared with the competing methods. To address the clinic problem of CBD stone detection, we develop the unique engineering solution with the collaboration between physicians and engineers to achieve the CBD stone diagnosis. In the future, we will apply the proposed method to different interdisciplinary fields of biomedical engineering such as [48], [51], and [52] to evaluate the generalization capability of the proposed method.
